# “I know exactly what I'm going into”: recommendations for pre‐nursing experience from an evaluation of a pre‐nursing scholarship in rural Scotland

**DOI:** 10.1002/nop2.23

**Published:** 2015-09-19

**Authors:** Annetta Smith, Michelle Beattie, Richard G. Kyle

**Affiliations:** ^1^ School of Health SciencesUniversity of Stirling (Highland Campus)Centre for Health ScienceInvernessUK; ^2^ School of NursingMidwifery and Social CareEdinburgh Napier UniversityUK

**Keywords:** Action research, nurse recruitment and retention, pre‐nursing experience, qualitative research, scholarship

## Abstract

**Aim:**

To develop a model of pre‐nursing experience from evaluation of a pre‐nursing scholarship for school pupils in Scotland.

**Design:**

Action research study.

**Methods:**

School pupils (*n* = 42) completed questionnaire surveys and participated in anecdote circles. Student nurses acting as pupil ‘buddies’ (*n* = 33) participated in focus groups. Descriptive quantitative data and thematic analyses of qualitative data were integrated across cohorts and campuses.

**Results:**

Ten recommended components of a model of pre‐nursing experience were identified: educational experience of: (1) face‐to‐face on‐campus teaching; (2) hands‐on clinical skills sessions; and (3) andragogy, practice exposure to (4) nursing language; (5) nurses’ emotional labour; (6) patients’ stories; (7) pupils socializing with buddies; (8) buddies planning placement activities; and (9) supporting pupils during placements. Academic attainment was not a central component of the model due to pupils’ need to (10) prioritize examined work for further/higher education entry.

## Introduction

Set in the context of global nursing shortages promotion of pre‐nursing experience has been proposed as a potential mechanism to aid nurse recruitment and selection internationally, especially in rural communities. Pre‐nursing experience can broadly be defined as an opportunity for individuals seeking entry to the nursing profession to gain insight into the life and work of a qualified or student nurse through, for example, observation of nursing practice in clinical environments or participation in nurse education on university or college campuses. Internationally, a range of approaches to pre‐nursing experience have been adopted by various healthcare, corporate and academic organizations, including: work experience, such as the Step into the NHS programme in the UK (NHS Careers, 2015); immersive overseas clinical placements provided by companies such as GapMedics to prospective nurses from the UK, USA, Canada and Australia in locations including Poland, Croatia, Tanzania and Thailand (GapMedics 2015); and nursing camps organized by academic institutions, especially in the USA (Drenkard *et al*. 2002, Daumer & Britson 2003, Matutina, 2008).

Despite the increased prominence of pre‐nursing experience and proliferation of alternative approaches, there is a dearth of evidence around the effects and effectiveness of different models of pre‐nursing experience and where outcomes are reported only weak description of the components of pre‐nursing experience that contribute to the realization of outcomes.

This paper addresses this knowledge deficit by reporting recommended components of pre‐nursing experience derived from evaluation of a 2‐year pilot pre‐nursing scholarship in remote and rural Scotland. In so doing, a model of pre‐nursing experience is proposed for replication and adaptation elsewhere.

## Background

### Pre‐nursing experience

In the UK, pre‐nursing experience has gained increased prominence following the UK Government's acceptance of recommendations in the Francis Inquiry into care failings at Mid‐Staffordshire NHS Trust (Francis 2013) that aspirant nurses work for a year as a healthcare assistant (HCA) prior to commencing undergraduate nurse education (Department of Health 2013). Health Education England (HEE) have subsequently established ‘pre‐nursing experience pilots’ for 150‐250 aspiring student nurses at a cost of £3·5 million (Hunt 2013). Despite this endorsement, the approach has been challenged by the UK Council of Deans of Health who question the limited evidence base supporting the scheme (Council of Deans of Health 2013). Importantly, recent longitudinal research in Scotland has shown that previous caring experience is associated with poorer academic and practice attainment (Kleebauer 2014). Evaluation results from pilot sites are not expected to report until 2018 (Health Education England 2014).

Academic organizations too have used a range of strategies to attract school leavers into nursing. Studies from the USA suggest pre‐nursing experience can promote and change high school pupils’ perception of the nursing profession. Drenkard *et al*. (2002) describe a Nursing Exploration Summer Camp which enabled twenty pupils aged 12‐15 years to experience nursing through hands‐on experience at local hospitals, attending classes in cardiopulmonary resuscitation (CPR) and interacting with university nursing students. After camp, students who considered nursing as a future career increased from 70% (*n* = 14)‐90% (*n* = 18) (Drenkard *et al*. 2002). Similarly, Daumer and Britson (2003) discuss a 4‐day nursing camp for high school pupils that incorporated job shadowing, CPR and first aid certification, lectures and social activities, that accrued credit for college entry. Following the experience, 90% of participants stated it ‘confirmed and strengthened their commitment to enter into nursing’ (Daumer & Britson 2003, p. 132). Despite evidence that initiatives, such as nursing camps, significantly increase school pupils desire to pursue a nursing career (Matutina 2008), these interventions and their evaluations have not been described in sufficient detail to enable adaptation.

Hence, there are limited descriptions and evaluations of pre‐nursing experience programmes from nursing camps in the USA to guide development of pre‐nursing interventions and no existing programmes in the UK that provide aspirant nurses with pre‐nursing experience through engagement with student nurses rather than HCA roles. Despite this, a study from Scotland suggests that nursing is not a first choice career among many penultimate and final‐year school pupils (Neilson 2008) and pupils described that work experience opportunities were of poor quality and requested opportunities to gain representative and realistic insight into nursing practice (Neilson & McNally 2010). Development and evaluation of programmes designed to promote nursing careers in Scotland is therefore appropriate and timely.

### Pre‐nursing scholarship

#### Rationale

The World Health Organisation has observed that ‘one of the most complex challenges is ensuring people living in remote and rural locations have access to trained healthcare workers’ (WHO 2010). Nurses represent the largest professional group in international healthcare systems and as such play a pivotal role in sustaining rural healthcare delivery. Attracting and retaining nurses in rural areas is therefore a priority for governments and healthcare organizations internationally. In Scotland (UK), the Government has acknowledged the challenges associated with rural healthcare delivery and the crucial role of the nursing workforce, especially in community settings, to maintain quality of care in the National Health Service (NHS) (Scottish Government 2008).

Educational interventions can support sustainability of rural healthcare. A recent systematic review identified that recruiting and training healthcare students in rural areas predicts return to these areas after training (Trépanier *et al*. 2012). Hence, by extension, evidence suggests that pre‐nursing experience that alerts aspirant nurses to the availability of training closer to home may increase recruitment and retention of healthcare workers in rural areas. In the light of this workforce need and evidence, the School of Health Sciences at the University of Stirling (between 2012–2014), working in partnership with local NHS Boards and education authorities, piloted a 9‐month ‘pre‐nursing scholarship’ (PNS) for penultimate and final‐year secondary school pupils (aged 15‐18 years) from remote and rural parts of the Scottish Highlands and Western Isles (Chisholm 2013).

#### Aim

The aims of the scholarship were to inspire school leavers to consider nursing careers, aspire to university‐level education and alert pupils to the availability of nurse education closer to home.

#### Delivery

The PNS was delivered in the University of Stirling's Highland Campus in Inverness and Western Isles Campus in Stornoway. The project was funded (£24,000) by NHS Education for Scotland (NES) with contributions from two local authorities serving the Highlands and Western Isles. Delivery costs, including pupils’ accommodation, catering, travel and social events during the residential week, totalled £14,700, equivalent to £350 (USD 530, EUR 495) per pupil who completed the scholarship (*n* = 42). Academic staff members time spent on project delivery, including organization of placements and delivery of taught components of the scholarship, represented an in‐kind contribution from the University was not apportioned to the project. Evaluation costs are not included in these totals.

#### Structure

**Figure 1:**
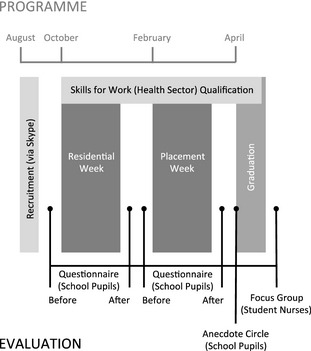
Pre‐nursing scholarship programme and evaluation.

The structure of the scholarship is illustrated in Figure 1 and comprised four core components:

- *Residential Week*: university experience over 5 consecutive days with overnight accommodation near campus. Structured activities included face‐to‐face and video conferenced lectures, clinical skills and planned evening social activities. Final‐year student nurses attended university experience and social activities.
- *Placement Week*: observational placement over four or five consecutive days in a NHS hospital and/or community setting close to the pupils’ home that focussed on care of older adults. School pupils were paired with and supported by a final‐year student nurse ‘buddy’. The buddy's practice mentor supervised the experience.
- *Qualification*: completion of a Skills for Work (Health Sector) qualification accredited by the Scottish Qualification Authority (SQA) over the 9 months of the scholarship;
- *Graduation*: event recognized school pupils successful completion of the scholarship attended by parents/carers, teachers and other education partners, academics, practice mentors and student nurse buddies.

#### Evaluation

Evaluation was embedded throughout the PNS (Figure 1). We have described elsewhere the *experiences* of involvement in the scholarship from the perspective of school pupils (Beattie *et al*. 2014) and student nurse buddies (Smith *et al*. 2015). In this paper we scale‐up and integrate our analysis across cohorts, campuses and data sources to derive recommendations for pre‐nursing experience that emerged from the evaluation.

#### Aim

To develop a model of pre‐nursing experience from evaluation of a pre‐nursing scholarship for school pupils in Scotland.

## The Study

### Design

Elliot's (1991) action research framework underpinned the evaluation. An action research approach was adopted due to the concurrent nature of the study and project delivery and to ensure that initial evaluation findings informed ongoing project delivery. Action research is an approach to enquiry that is participatory and grounded in experience (Reason & Bradbury 2006). Herr and Anderson (2005) describe the double goals of action research as being concerned with both action (improvement of practice) and research (creating valid knowledge about practice). Hence, two evaluation cycles associated with each of the two PNS cohorts were conducted. Changes were made to the programme as a result of initial findings after the first cycle. No changes were made to evaluation methods during the programme.

### Method

#### Participants

Participants in the scholarship in both years included: school pupils in their final or penultimate year of secondary education in Scotland (Secondary 5 or Secondary 6) from 15 schools across the Scottish Highlands and Western Isles; final (third) year student nurse ‘buddies’ who were paired with pupils throughout the scholarship; and practice mentors who supported student nurse buddies during the placement week (Smith *et al*. 2015).

#### Data collection

A mixed‐methods approach to data collection was adopted (Table 1). Quantitative and qualitative data were collected from school pupils in each cohort (i.e. 2012/13, 2013/14) using a questionnaire survey and anecdote circles and focus groups were held with student nurse buddies (Figure 1).

**Table 1:** Data sources and response/participation rates

Participants	Data source^a^	Campus				Total	
		Highland		Western Isles			
		*n*	%^b^	*n*	%	*n*	%
School pupils	Questionnaire returns	104	92·9	44	78·6	148	88·1
	Residential week	55	98·2	27	96·4	82	97·6
	*Before*	27	96·4	13	92·9	40	95·2
	*After*	28	100	14	100	42	100
	Placement week	49	87·5	17	60·7	66	78·6
	*Before*	23	82·1	9	64·3	32	76·2
	*After*	26	92·9	8	57·1	34	81·0
	Anecdote circles	28	100	14	100	42	100
Student nurses	Focus groups	25	96·2	8	100	33	97·1

a Data for the 6 (12·5%) pupils who withdrew from the scholarship have been excluded.

b For questionnaires, % represents the response rate; for anecdote circles and focus groups, % represents participation rate.

#### School pupils

##### Questionnaires

School pupils completed paper‐based questionnaires before and after the Residential Week and Placement Week (*n* = 148; 88·1% response rate; Table 1). Questionnaire structure and content is shown in Table 2.

**Table 2:** Questionnaire content

Time point	Residential week		Placement week	
	Question	Items/Response options	Question	Response options
Before *Hopes, Plans and Expectations*	Was Skype a good way to interview you for your place on the scholarship?	2 items, Yes/No and open explanation	What do you expect to do during your Placement Week?	1 item, open
	What are you most looking forward to during the Residential Week?	1 item, open	What are you most looking forward to during the Placement Week?	1 item, open
	What do you hope to get out of the Residential Week?	1 item, open	What do you hope to get out of the Placement Week?	1 item, open
After *Evaluation*	How would you rate the following aspects of the Residential Week?	12 items, 5‐point Likert scale: very poor to very good (Table 6)	To what extent do you agree or disagree with the following statements?	8 items, 5‐point Likert scale: strongly disagree to strongly agree (Table 6)
	If you answered {satisfactory, poor, Very poor} or {very good or good} please say why?	2 items, open	How has/hasn't the Placement Week… a) given you a better understanding of what nurses do? b) changed your view about what nursing is all about? c) given you a better understanding of the skills that you would need to be a nurse? d) made you feel that you are in a better position to make a decision about whether nursing is right for you?	4 items, open
	What did you enjoy {most/least} about the Residential Week?	2 items, open	What did you enjoy {most/least} about the Placement Week?	2 items, open
	Did you get out of the Residential Week what you hoped to get out of it?	2 items, Yes/No and open explanation	Did you get out of the Placement Week what you hoped to get out of it?	2 items, Yes/No and open explanation
	Do you think the amount of information given about nursing during the Residential Week was…	1 item, 3‐point Likert scale: too little/about right/too much	Do you think the amount of time spent on Placement was…	1 item, 3‐point Likert scale: too little/about right/too much
Before and After both Residential and Placement Week	Please tick the statements that best apply to you.	2 items, 5‐point Likert scale: ‘I definitely don't want a career in nursing/another healthcare profession’ to ‘I definitely want a career in nursing/another healthcare profession’ (Table 4).	What do you plan to do when you leave school?	1 item, 7 categories derived from annual School Leaver Destination Return (SDLR) conducted by Skills Development Scotland (Table 4)

##### Anecdote circles

School pupils (*n* = 42) participated in an anecdote circle on the day of their Graduation (Table 1). Our adaptation of the anecdote circle technique is described elsewhere (Beattie *et al*. 2014). Briefly, each pupil participated in storytelling around their experiences of three PNS components; residential week, placement week and qualification. Data included both verbatim transcripts of pupils’ audio‐recorded discussion and written comments.

#### Student nurse buddies

##### Focus groups

Final‐year student nurse buddies (*n* = 33) who supported pupils during the PNS participated in a focus group at the end of each cohort. Our focus group approach has been described elsewhere (Smith *et al*. 2015). Briefly, hour‐long focus groups were lightly facilitated to encourage a shared understanding of the buddying experience to emerge. Data comprised verbatim transcripts of audio‐recorded discussion. Findings around student nurse buddies’ experiences and perceived outcomes of involvement in the scholarship have been reported elsewhere (Smith *et al*. 2015).

### Analysis

Quantitative data from questionnaires were entered into SPSS version 21 (SPSS, Inc, Chicago, IL, USA). Descriptive statistics were calculated for sample characteristics (i.e. gender, age, school year, educational aspirations and career intentions) and scholarship outcomes (pupil satisfaction and statements assessing scholarship experiences following the placement week) and reported as n (%). Due to the relatively small sample size, statistical tests were not conducted to assess change over time and hence are also reported descriptively. Qualitative data from questionnaires, written comments from anecdote circles and verbatim transcripts of anecdote circle and focus group discussion were analysed thematically. Each data source was first analysed separately by cohort and campus and subsequently integrated to meet the aim of the evaluation and draw recommendations for pre‐nursing experience from evaluation of the pre‐nursing scholarship pilot as a whole.

Analysis was conducted through a series of data workshops. Prior to each data workshop, co‐authors read the relevant data independently to derive themes which were then agreed through an iterative process of refining themes through discussion and constant comparison within and between data sources. In turn, these data workshops: (1) examined school pupils’ experiences of the PNS reported through written comments and transcripts from anecdote circle discussion (Beattie *et al*. 2014); (2) explored student nurse buddies experiences of the PNS through focus groups transcripts (Smith *et al*. 2015); (3) integrated data sources across cohorts and campuses to derive recommendations for pre‐nursing experience and develop a replicable and adaptable model.

This third analysis stage was underpinned by Donabedian's (1980) structure, process and outcome framework, adapted through the addition of a fourth component – recommendation. A matrix was created (available from the corresponding author on request) with the following columns and populated by all co‐authors through discussion at the third data workshop:

- *Structure*: described the contextual elements of the scholarship programme.
- *Processes*: described the activities of the scholarship that realized anticipated or unanticipated outcomes.
- *Outcomes*: were the expected or unexpected results from specific processes in particular structures.
- *Recommendations*: identified the potentially transferrable lessons from the PNS that contributed to realization of perceived outcomes.

Reporting of findings begins with a brief overview of the sample, followed by the outcomes of the pre‐nursing scholarship. Each Recommendation for pre‐nursing experience is then presented in turn and finally integrated to present a model of pre‐nursing experience.

### Ethics

The study was approved by a University Research Ethics Committee. All participants provided written informed consent before participating in an anecdote circle or focus group. Anonymity has been assured through the use of a unique participant identifier indicating cohort and campus (e.g. Pupil 1).

### Results sample

The sample included 42 school pupils and 33 student nurse buddies (Table 3). Characteristics of the school pupils who completed and withdrew from the scholarship are presented in Table 4. Of the pupils completing the PNS, all except one was female (98%) and 69·0% were in their final year of secondary education. At the start of the PNS, 71·4% expressed a probable or definite desire to pursue a nursing career.

**Table 3:** Pre‐nursing scholarship completion rates by cohort and campus

Cohort	Campus						Total		
	Highland			Western Isles					
	Commenced	Completed		Commenced	Completed		Commenced	Completed	
	*n*	*n*	%	*n*	*n*	%	*n*	*n*	%
2012/13	12	12	100	10	7	70·0	22	19	86·4
2013/14	17	16	94·1	9	7	77·8	26	23	88·5
Total	29	28	96·5	19	14	73·7	48	42	87·5

**Table 4:** Pre‐nursing scholarship participant characteristics

	Completed (*n *=* *42)		Withdrew (*n *=* *6)	
	*n*	%	*n*	%
Gender				
Male	1	2·4	–	–
Female	41	97·6	6	100
Age				
15	4	9·5	1	16·7
16	22	52·4	3	50·0
17	16	38·1	1	16·7
18	–	–	1	16·7
School Year				
Secondary 5	13	31·0	2	33·3
Secondary 6	29	69·0	4	66·7
Educational Aspirations^a^				
Do voluntary work	3	7·1	–	–
Paid employment	1	2·4	–	–
Go on to work‐based training	2	4·8	–	–
Go to college	3	7·1	1	16·7
Go to university	36	85·7	2	33·3
Go travelling/take a gap year	4	9·5	–	–
Not sure yet	2	4·8	2	33·3
Career intentions				
Nursing				
I definitely don't want a career in nursing	–	–	–	–
I probably don't want a career in nursing	1	2·4	–	–
I am not sure if I want a career in nursing	5	11·9	3	50·0
I probably want a career in nursing	11	26·2	–	–
I definitely want a career in nursing	19	45·2	2	33·3
Missing	6	14·3	1	16·7
Other health‐related professions				
I definitely don't want a career in another health profession	–	–	–	–
I probably don't want a career in another health profession	2	4·8	–	–
I am not sure if I want a career in another health profession	12	28·6	4	66·7
I probably want a career in another health profession	9	21·4	–	–
I definitely want a career in another health profession	4	9·5	1	16·7
Missing	15	35·7	1	16·7

a Pupils could select more than one option for this question.

### Outcomes

After the PNS there was an increase in the percentage of pupils indicating that they ‘definitely’ wanted to pursue a nursing career (54·5%, *n* = 18–63·6%, *n* = 21). Most pupils strongly agreed (79·4%) that the PNS had put them in a ‘better position’ to decide whether nursing was right for them (Table 5). Pupils written comments also indicated that for some it ‘confirmed’ (P26) their choice or resulted in increased ‘confidence’ (P25) or ‘certainty’ (P9,36) in their decision:

**Table 5:** Placement week statements

Statement	Strongly agree		Agree		Neither Agree or Disagree		Disagree		Strongly Disagree	
	*n*	%	*n*	%	*n*	%	*n*	%	*n*	%
I had a good relationship with my buddy	30	88·2	4	11·8	–	–	–	–	–	–
I had a wide variety of experiences during my placement week	18	52·9	15	44·1	1	2·9	–	–	–	–
I got the opportunity to speak to patients during my placement week	22	64·7	11	32·4	1	2·9	–	–	–	–
I was made to feel welcome by the nursing staff during my placement week	30	88·2	4	11·8	–	–	–	–	–	–
I have a better understanding of what nurses do after the placement week	28	82·4	6	17·6	–	–	–	–	–	–
My placement week has changed my view of what nursing is all about	13	38·2	14	41·2	6	17·6	1	2·9	–	–
I have a better understanding of the skills I would need to be a nurse after my placement week	25	73·5	9	26·5	–	–	–	–	–	–
I feel in a better position to make a decision about whether nursing is right for me after my placement week	27	79·4	5	14·7	2	5·9	–	–	–	–

I am in a better position to make a decision because it has made me feel more strongly about going into nursing. It has made me want to be a nurse even more. (P2)

It confirmed my choice for me as I really enjoyed the time spent on placement. (P26)

Having the experience makes me more confident in my career choice. (P25)

I now know for sure I want to be a nurse. (P21)

### Recommendations for pre‐nursing experience

Ten recommendations for pre‐nursing experience were identified through evaluation (Box 1).

Box 1 **Recommendations for pre‐nursing experience**

1

**Table:** 

Educational experiences	
1.	Face‐to‐face on‐campus teaching
2.	Hands‐on clinical skills sessions
3.	Andragogical underpinnings
Practice exposures	
4.	Nursing language
5.	Nurses’ emotional labour
6.	Patients’ stories
Buddying process	
7.	Pupils socializing with buddies
8.	Placement activities planned by buddies
9.	Pupils supported through buddies
Academic attainment	
10.	Prioritize examined work for further/higher education entry

### Educational experiences

The residential week provided pupils with insight into the experience of being a student nurse; enabling them to assess their suitability for student life and their desire to be a student nurse.

Three inter‐connected educational experiences are therefore recommended to enable aspirant nurses to gain insight into life as a student nurse:

#### Face‐to‐face on‐campus teaching

Satisfaction ratings revealed a preference for face‐to‐face teaching rather than remotely delivered content via video‐conferencing (Table 6). Campus teaching was perceived to be essential to helping pupils to feel like a student nurse and provided a taster for their future nurse education experience.

**Table 6:** Residential week satisfaction^a^

Content	Cohort																			
	2012/13										2013/14									
	Very good		Good		Satisfactory		Poor		Very poor		Very good		Good		Satisfactory		Poor		Very poor	
	*n*	%	*n*	%	*n*	%	*n*	%	*n*	%	*n*	%	*n*	%	*n*	%	*n*	%	*n*	%
Welcome and Introduction	16	84·2	3	15·8	–	–	–	–	–	–	16	69·6	7	30·4	–	–	–	–	–	–
Initial meeting with my buddy^b^	17	89·5	2	10·5	–	–	–	–	–	–	14	73·7	4	21·1	1	5·3	–	–	–	–
Introduction to professional/ethical issues^c^	11	57·9	8	42·1	–	–	–	–	–	–	9	45·0	11	55·0	–	–	–	–	–	–
Introduction to library skills^d^	4	22·2	11	61·1	3	16·7	–	–	–	–	1	10·0	2	20·0	3	30·0	3	30·0	1	10·0
STEER Training (via VC])^e^	3	15·8	5	26·3	7	36·8	4	21·1	–	–	–	–	–	–	–	–	–	–	–	–
Interviewing technique^f^	–	–	–	–	–	–	–	–	–	–	9	56·3	5	31·3	2	12·5	–	–	–	–
Cardiovascular system (via VC in 2012/13)	11	57·9	6	31·6	2	10·5	–	–	–	–	14	60·9	7	30·4	2	8·7	–	–	–	–
Clinical skills training	16	84·2	3	15·8	–	–	–	–	–	–	23	100	–	–	–	–	–	–	–	–
‘So, you want to be a nurse?’ day^g^	11	57·9	8	42·1	–	–	–	–	–	–	8	50·0	8	50·0	–	–	–	–	–	–
Accommodation	8	42·1	1	5·3	6	31·6	–	–	4	21·1	–	–	13	56·5	5	21·7	2	8·7	3	13·0
Catering	15	78·9	4	21·1	–	–	–	–	–	–	8	34·8	14	60·9	1	4·3	–	–	–	–
Social activities	16	84·2	3	15·8	–	–	–	–	–	–	15	65·2	8	34·8	–	–	–	–	–	–
Life sciences industry session^h^	–	–	–	–	–	–	–	–	–	–	22	95·7	1	4·3	–	–	–	–	–	–

a Disaggregated by cohort due to changes to programme between years.

b Four pupils in 2013/14 cohort did not answer this question.

c Three pupils in 2013/14 cohort did not answer this question.

d One pupil in 2012/13 cohort and 6 pupils in 2013/14 cohort did not answer this question. Only offered in Highland in 2013/14 cohort.

e Not offered in 2013/14 cohort.

f Only offered in Highland in 2013/14 cohort.

g Only offered in Highland in 2012/13 and 2013/14 cohort.

h Not offered in 2012/13 cohort.

#### Hands‐on clinical skills sessions

Satisfaction with clinical skills sessions rated high and all pupils in the 2013/14 cohort across both campuses rated this session ‘very good’ (Table 6). Indeed, the academic facilitators were frequently singled out for praise, exemplifying the positive impact this content had on pupils’ educational experience. Pupils particularly welcomed the contrast with lecture‐based content:

The clinical skills training was really fun because we could do something not just sit and listen (P17)

#### Andragogy

School pupils valued the andragogical philosophy that underpinned their time on campus and the way this was demonstrated through subject relevance, respect and relationships. Pupils valued the ease of their developing relationship with academic staff and student nurse buddies. For example, pupils noted:

I got to experience learning in an adult environment and get treated like one and also got to know exactly what nursing involves. (P14)

The Buddies were very friendly and kind to us. They treated us like adults which we were happy about too. (P20)

### Practice exposures

The placement week exposed school pupils to the totality, complexity and reality of nursing practice. Following the placement week, all pupils ‘strongly agreed’ or ‘agreed’ that they had a better understanding of nurses’ roles and skills (Table 5). In particular, placements had ‘opened their eyes’ to the diversity and complexity of nursing practice:

My placement has given me a better understanding of what nurses do as I was unaware of just how much the nurses do. (P29)

It showed me the range of skills you need to have from dealing with little children to the elderly. (P11)

It has shown me that they do a lot more than I thought they did. (P41)

Relational skills such as ‘communication’ (P1,5,7,9,19,21,35,37,39,41), ‘listening’ (P7,19), ‘teamwork’ (P35,39) were most frequently highlighted and ‘good co‐operation’ (P37), ‘decision‐making’ (P29), ‘confidentiality’ (P11) and ‘responsibility’ (P5) noted. Personal attributes becoming of a nurse were also cited including ‘remain[ing] calm’ (P10), ‘empathy’ (P19) and ‘confidence’ (P29) as well as having a ‘positive attitude’ (P21) and ‘caring nature’ (P5,41). For example, pupils typically wrote:

We saw that nurses require a lot of skills both clinically and emotionally. Communication and teamwork are vital. Before placement I didn't realise how many skills nurses needed. (P39)

It showed me that to be a nurse you have to be a kind, loving person who feels as if they have to have a vocation to care for people. (P43)

Placement experience enabled school pupils to determine their ability and suitability for nursing either affirming their career choice or deciding once exposed to the reality of nursing that it was not the right career for them:

It made me realise I was quite confident while speaking with the patients and that I really enjoyed it. (P5)

Placement has given me a better understanding of the skills I would need as a nurse as I practiced some of them such as communicating with patients. (P26)

It helped me to realise that I can handle difficult situations well. (P8)

Notably, one pupil developed a realization that the demands of a nursing career required considerable effort:

It has made me realise just how difficult nursing is and that if I want to pursue a career in it I need to work harder (P29)

Three inter‐connected practice exposures are therefore recommended to enable aspirant nurses to gain insight into the totality, complexity and reality of the nursing role.

#### Nursing language

Following placement most pupils strongly agreed (88·2%, *n* = 30) that they ‘had been welcomed by’ and ‘had a good relationship with their buddy’ (Table 5). Through this relationship rapid socialization into nursing occurred through exposure to nursing language. Pupils written and spoken comments were replete with phrases nurses typically use, such as ‘doing the obs’. Hence, expressing an affinity with their future professional identity.

#### Patients’ stories

Before placement, patient interaction was among the most eagerly anticipated: ‘I am really looking forward to interacting with patients’ (P26). Following placement, all bar one pupil (97·1%, *n* = 33) strongly agreed or agreed that they ‘got the opportunity to speak with patients (Table 5) and this emerged as the most enjoyable part of the placement week:

The part that I most enjoyed about placement would be being able to communicate with the patients in the ward and having conversations with them when I wasn't busy. (P26)

I mostly enjoyed talking to the different patients and hearing about their experiences in the hospital and their lives. (P12)

I enjoyed going out to visit the patients’ houses and being able to see how they would be taken care of. Being able to see how other people, who are unwell, live around their ailments. (P2)

#### Nurses’ emotional labour

Opportunities to speak with patients and to listen to individuals’ life stories and illness narratives, were transformative, shifting pupils’ perceptions of older adults and specific diseases, such as cancer and dementia. Indeed, hearing sad stories and particularly those involving death and bereavement affirmed pupils’ desire (or otherwise) to pursue a nursing career.

### Buddying

Pupils and buddies described how ‘buddying’ was a crucial PNS component. Following the residential week, the initial meeting with the buddies obtained a high satisfaction rating, especially among the 2012/13 cohort (Table 6). Indeed, after this week, pupils commented that they ‘felt very comfortable with the buddies’ (P16) and that they ‘loved having the buddies – great help and support’ (P6). As a result, prior to the placement week many pupils were most looking forward to ‘working alongside my buddy’ (P39) and hoped that doing so would give them ‘a better insight into nursing’ (P25) and particularly ‘an understanding of the workload of being a student from my buddy’ (P28). Another pupil noted that they were most looking forward to ‘having the chance to work with a third year student and getting to know what is expected (P23). Following placement, all students stated they got out of the placement week what they hoped (*n* = 34, 100%). The buddying process was found to be an essential element of the scholarship as it facilitated vicarious experience:

Being able to get a first‐hand insight into nursing I now know that hard work is extremely important and essential to the job. (P28)

As I have now seen what it's like in person I understand it more now it really is a great job. (P31)

It gave me a much better understanding as instead of being told what I would do if I was to go into nursing I got to see it all in action on the ward. (P26)

The perceived authenticity of the placement experience accessed through vicarious experience also emerged in pupils’ comments. Frequently, pupils noted that time on placement had revealed the reality of nursing practice:

It has given me a better understanding by seeing what actually happens on the wards and what nurses have to deal with on a day‐to‐day basis. (P7)

It gave me the opportunity to talk to patients/nurses about their experiences and also to see what exactly the nurses do. (P9)

“I know exactly what I'm going into” (P24)

#### Socializing

Planned social activities, such as bowling, were rated highly by pupils and students (Table 6). However, while arranged social activities were enjoyed by pupils, most valued were the friendships that were established through informal socializing between buddies and pupils during the residential week. Following evaluation of the first cohort, several pupils expressed a desire for fewer structured activities in favour of more time for informal interaction with buddies. However, after making this change for the second scholarship cohort the proportion of pupils reporting that social activities were ‘very good’ decreased from 84·2% (*n* = 16) to 65·2% (*n* = 15), suggesting that the degree of structure imposed around social activities requires to be responsive to each cohort and is perhaps best negotiated with pupils at the start of the residential week.

Three aspects of the buddying process are therefore recommended to enable aspirant nurses to access vicarious experience of nursing:

#### Placement activities planned by buddies

Responsibility for planning the pupils’ placement was devolved to the buddy supported from their practice mentor. While some student buddies’ found this role challenging, they recognized that it provided an opportunity to ‘step up’ and rehearse future mentoring roles. Teaching and supporting pupils in this way affirmed buddies’ nursing knowledge and competence.

#### Pupils supported through buddies

Placement support required buddies to display high levels of situational awareness and to empathetically respond to pupils’ potential emotional distress, for example, after learning about the death of a patient. Pupils shared how they felt supported in practice.

### Academic attainment

#### Prioritize examined work for further/higher education entry

School pupils did not perceive completion of the qualification as a crucial aspect of the pre‐nursing scholarship. Rather, pupils reported that some of the theoretical content was burdensome and several suggested that the qualification had diverted attention from pre‐requisite school work required to secure entry to further or higher education (FE/HE).

### Model of pre‐nursing experience

**Figure 2:**
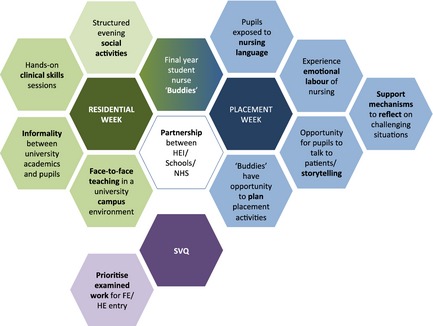
Pre‐nursing experience model.

Figure 2 presents a model of pre‐nursing experience that integrates the ten recommendations identified from evaluation of the PNS. Each recommendation is presented on a hexagon and arranged such that recommendations interface with other related recommendations, as well as core scholarship components (i.e. residential week, placement week) and underpinning processes (i.e. buddying) with which they are associated. As our analysis suggested completion of a qualification during the scholarship was not a priority and is separated in the model.

## Discussion

Evaluation of the pre‐nursing scholarship identified that participation in pre‐nursing experience enabled school pupils to make an informed decision about pursuing a nursing career. These findings confirm research from the USA (Drenkard *et al*. 2002, Daumer & Britson 2003) that pre‐nursing experience supports nurse recruitment and retention as aspirant nurses assess their suitability for nursing. However, our study extends this evidence by outlining the educational experiences, practice exposures and underpinning processes that contributed to the realization of these outcomes. Our findings suggest that pre‐nursing experience should involve both ‘on campus’ and ‘in practice’ components and is enhanced through peer mentorship that enables vicarious educational experiences and practice exposures.

Vicariously experiencing the life and work of a student nurse through relationships with final‐year student nurse buddies was a central element of the scholarship. Both, informal off campus activities and campus shared lectures and clinical skills sessions, were particularly valued. Echoing findings from evaluations of nursing summer camps in the USA (Daumer & Britson 2003), practicing clinical skills aided pupils’ assessment of their suitability for nursing practice. Face‐to‐face teaching in a university campus environment and hands‐on clinical skills sessions delivered in accordance with andragogical principles were recommended educational experiences integral to pre‐nursing experience.

Pairing pupils with buddies exposed pupils to patients’ lives and the emotional labour of nursing. Daumer and Britson (2003) similarly found that nursing camp participants rated job shadowing highly and Norman *et al*. (2008) identified that a beneficial component of a nursing cadet scheme was to provide students with a realistic picture of nursing. Counter to Culley and Genders (2003) conclusion from a British cadet scheme for nursing that cadets under 18 years old as too immature to cope with the emotional demands of dying and acutely ill patients, our study found that exposure to emotional labour and particularly vicarious experience of sadness through patients’ life stories (Beattie *et al*. 2014) was supported through relationships with student nurse buddies and affirmed school pupils desire to nurse. Hence, pupils perceived that the scholarship provided a realistic insight into nursing practice. Unrealistic expectations of the nursing role have been found to be a key factor in nursing attrition (Norman *et al*. 2008). Thus, the opportunity to experience the emotional labour of nursing both first‐hand and vicariously through student nurse buddies potentially aids recruitment and retention of the nursing workforce and may support selection through provision of opportunities for aspirant nurses to de‐select nursing. At least one pupil (2·4%) shared that the scholarship had helped them to determine that nursing was not the right career choice for them (Beattie *et al*. 2014). Although caution should be exercised over extrapolation from a small‐scale pilot, if 2·4% of the 2015–2016 intake of student nurses in Scotland (*n* = 3038) (Scottish Government, 2015) obtained similar pre‐nursing experience, 73 nursing students may decline nursing as a career choice prior to commencing a programme, releasing places to other aspirant nurses. However, further research is required to assess the longer term impact of participation in the scholarship on selection and retention of student nurses, as well as how scholarship participants transition into pre‐registration nursing programmes (school pupils) and clinical practice (student nurse buddies).

Importantly, the scholarship's aim to alert pupils to the availability of nurse education closer to home was reinforced by a positive experience at their local campus. Recruitment and retention of health professionals to rural areas is known to be related to origin (Lea *et al*. 2008, Warburton *et al*. 2014) and positive educational placements (Walker *et al*. 2012) and being educated close to home influences where some nurse graduates seek work following graduation (McKenna *et al*. 2010).

Partnering, although not explicitly identified by participants, occupies a prominent position in the proposed model and has been found by others to be a key facilitator of positive pre‐nursing experiences (Daumer & Britson 2003, DeLapp *et al*. 2008). Negotiation was required between university providers of nurse education and local NHS Boards to facilitate access to placements and between Local Authority Education Departments and secondary schools to secure support for pupils to participate in the programme. For example, agreement was required between all partners to minimize disruption to pupils’ and students’ academic progress and practice placements.

The qualification was not perceived to be important for the pre‐nursing experience because pupils indicated that they needed to prioritize other examined work required for entry to further or higher education rather than the qualification that had limited currency. Pupils were generally critical of the need to undertake the qualification and it did not directly influence their decision about nursing careers. Participants in nursing summer camps in the USA that included accredited CPR and first aid training valued this certification (Daumer & Britson 2003) and it may be that this approach to accreditation would be more beneficial in future initiatives.

### Limitations

The main limitation is the relatively small number of students and pupils involved in the pilot. However, integration of data sources from multiple sources mitigates the risk of bias and enables transferrable recommendations to be made. Due to limitations of time and resources, data collection was not conducted with practice mentors.

## Conclusion

This is the first pre‐nursing scholarship to be developed and evaluated in the UK and we identify ten recommendations and propose a model to inform the design and delivery of similar future programmes. The study is timely given the prevailing policy around pre‐nursing experience in the UK, the relative paucity of robust evidence around effective approaches to enhance nurse recruitment, selection and retention, particularly in Scotland (Rodgers *et al*. 2013) and the enduring international challenge of rural nurse recruitment. The PNS provided an effective model by aiding pupils’ personal assessment of their suitability and desire for nursing. This evaluation suggests that alternative approaches that require aspirant nurses to spend time working in a different role as an HCA (Department of Health 2013, Francis 2013) may not provide the realistic insight into (student) nursing that was valued by pre‐nursing scholarship participants. Moreover, despite previous suggestions that pre‐nursing experience should be standardized (Culley & Genders 2003), evaluation of the pre‐nursing scholarship revealed the importance of change over time in response to each cohort and evaluation cycle, as well as adaptation to local care contexts. Our research suggests that future approaches to pre‐nursing experience in support of nurse recruitment and retention strategies should incorporate a degree of flexibility rather than implementing singular solutions for universal application.

## Conflict of interest

No conflict of interest has been declared by the authors(s).

## Author contributions

All authors have agreed on the final version and meet at least one of the following criteria [recommended by the ICMJE (http://www.icmje.org/recommendations/)]:

- substantial contributions to conception and design, acquisition of data, or analysis and interpretation of data;
- drafting the article or revising it critically for important intellectual content.
